# Supplementary material for: Carrier-Free Cellular Transport of CRISPR/Cas9 Ribonucleoprotein for Genome Editing by Cold Atmospheric Plasma
Source: Biology (Basel). 2021 Oct 13;10(10):1038. doi: 10.3390/biology10101038 (PMC8533602; doi:10.3390/biology10101038)
Supplement: Supplementary file 1 [file biology-10-01038-s001.zip › biology-1378506-supplementary.pdf]

Supporting Information

Carrier-free Cellular Transport of CRISPR/Cas9 Ribonucleoprotein for Genome Editing by Cold Atmospheric Plasma

Haodong Cui, Min Jiang, Wenhua Zhou, Ming Gao, Rui He*, Yifan Huang, Paul K. Chu and Xue-Feng Yu**

Cui, H.; Jiang, M.; Dr. Zhou, W.; Gao, M.; Dr. He, R.; Prof. Huang, Y.; Prof. Yu, X.-F.

Materials Interfaces Center,

Shenzhen Institute of Advanced Technology,

Chinese Academy of Sciences, Shenzhen 518055, China

E-mail: wh.zhou@siat.ac.cn, rui.he1@siat.ac.cn, xf.yu@siat.ac.cn

Cui, H.; Dr. Zhou, W.; Gao M.; Prof. Huang, Y.; Prof. Yu, X.-F.

University of Chinese Academy of Sciences, Beijing 100049, China

Prof. Chu, P. K.

Department of Physics, Department of Materials Science and Engineering, and Department of Biomedical Engineering,

City University of Hong Kong,

Tat Chee Avenue, Kowloon, Hong Kong, China

Keywords: Cold atmospheric plasma, CRISPR/Cas9, Carrier-free transport, Genome editing.

Table S1. Uptake and nuclear import efficiency of Cas9sg in MCF-7 cells *via* CAP transport and Lipofectamine transfection.

**Groups**	**Uptake efficiency (%)**	**Nuclear import efficiency (%)** [a)]
Control	22.1 ± 6.7	8.7 ± 3.5
CAP transport [b)]	88.9 ± 2.4	65.9 ± 4.2
Lipofectamine transfection	83.1 ± 2.7	72.9 ± 2.8

[a)] The nuclear import efficiency of Cas9sg-488 is determined at 24 h after CAP transport or Lipofectamine transfection.

[b)] Cells are exposed to CAP for 80 s.

Table S2. Comparison of Liopfectamine-mediated Cas9sg transfection.

References	HEK293 normal cell			References	MCF-7 tumor cell		
	% Indel	Cell Seeding Numbers/ well (10^3)	Concentration of Cas9-sgRNA complexes		% Indel	Cell Seeding Numbers/ well (10^3)	Concentration of Cas9-sgRNA complexes
[S1]	75	120	0.5 μg	[S1]	8	144	0.5 μg
[S3]	36-38	10	1.1 μg	[S2]	51.4	100	2 μg
This study	21.7	50	0.5 μg	This study	30.2	50	0.5 μg

Table S3. Comparison of carrier-free transportation of Cas9sg *via* Electroportation and CAP transport.

References	HEK293 normal cell			References	MCF-7 tumor cell		
	% Indel	Cell Seeding Numbers/ well (10^3)	Concentration of Cas9-sgRNA complexes		% Indel	Cell Seeding Numbers/ well (10^3)	Concentration of Cas9-sgRNA complexes
Electro-[S4]	88	100	2 μg	Electro-[S1]	22	200	24 μg
Electro-[S5]	5-65	350	40 μg	-	-	-	-
This study	21.7	50	0.5 μg	This study	30.2	50	0.5 μg

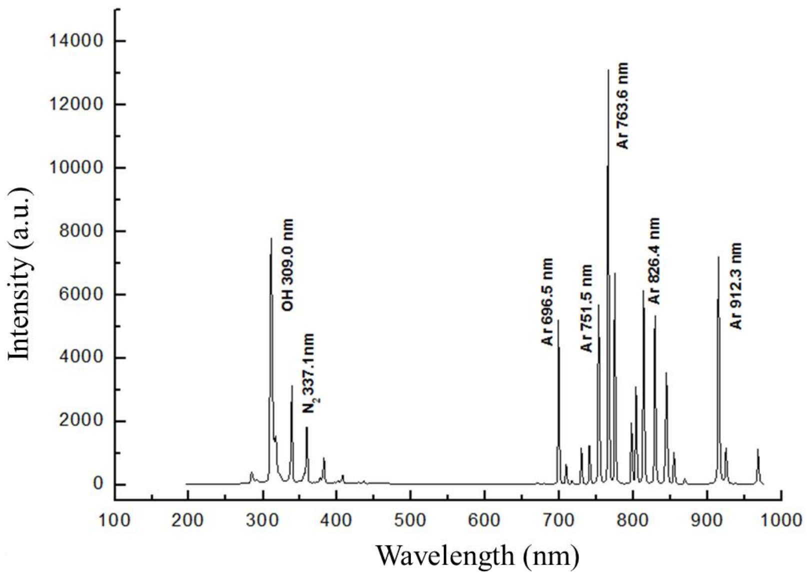

Figure S1. Representative optical emission spectra of the argon plasma.

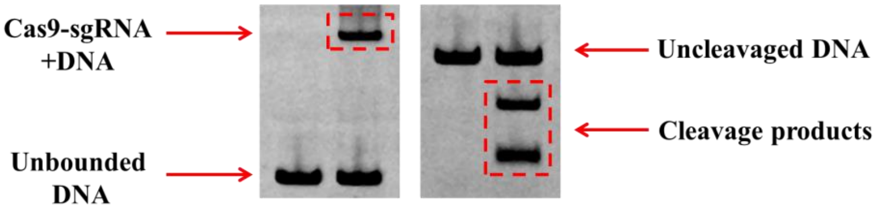

Figure S2. Binding/cleavage activities of Cas9 proteins: Cas9 protein (100 nmol L^{-1}) and sgRNA (200 nmol L^{-1}) are incubated together in 1×NEBuffer 2 for 30 min at 37 ℃ and then Cy5-labeled target DNA with a concentration of 150 nmol L^{-1} is added for another 30 min. The binding and cleavage activities of Cas9 protein are analyzed by 6% native PAGE. 6× native loading buffer and 10× SDS loading buffer are used to reveal the binding and cleavage activities respectively.

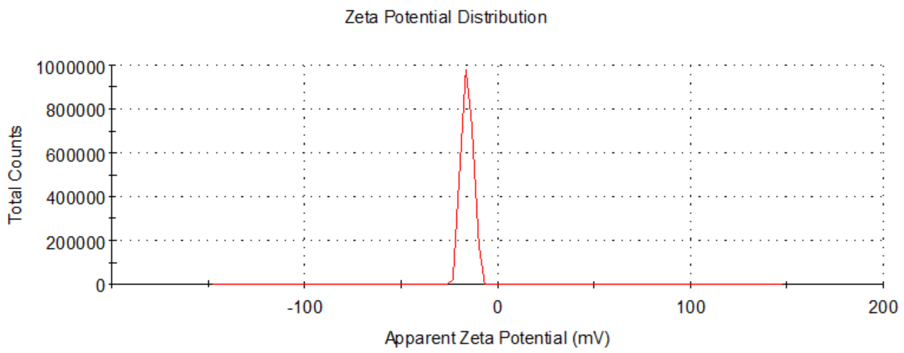

Figure S3. Zeta potentials of Cas9sg in 1× NEBuffer 2.

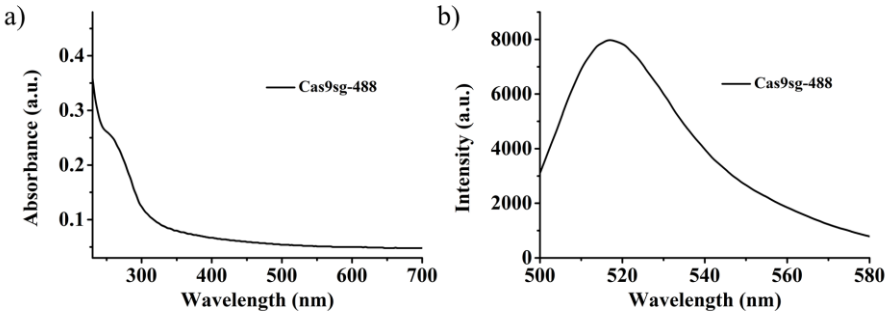

Figure S4. (a) The absorbance and (b) Fluorescence spectra of Cas9sg-488.

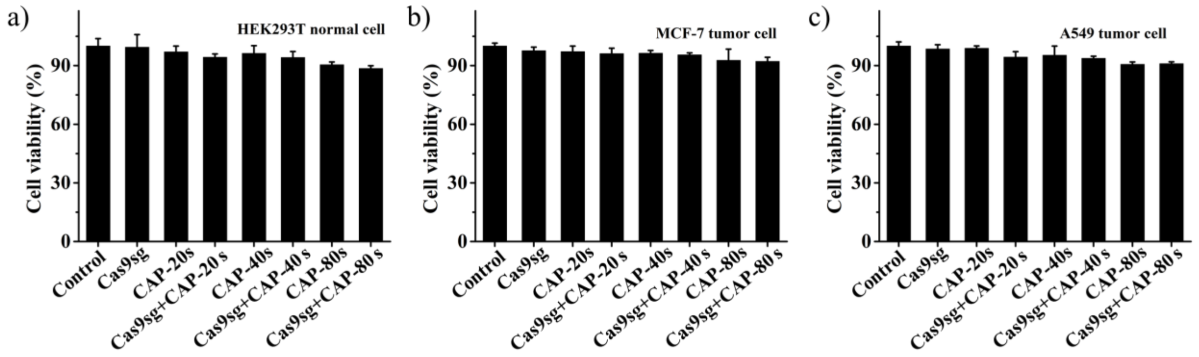

Figure S5. Cell viability of (a) HEK293T, (b) MCF-7, and (c) A549 cells for 24 h after the CAP treatment with or without Cas9sg determined by MTT assays. The data are shown as mean ± SD (*n*=5).

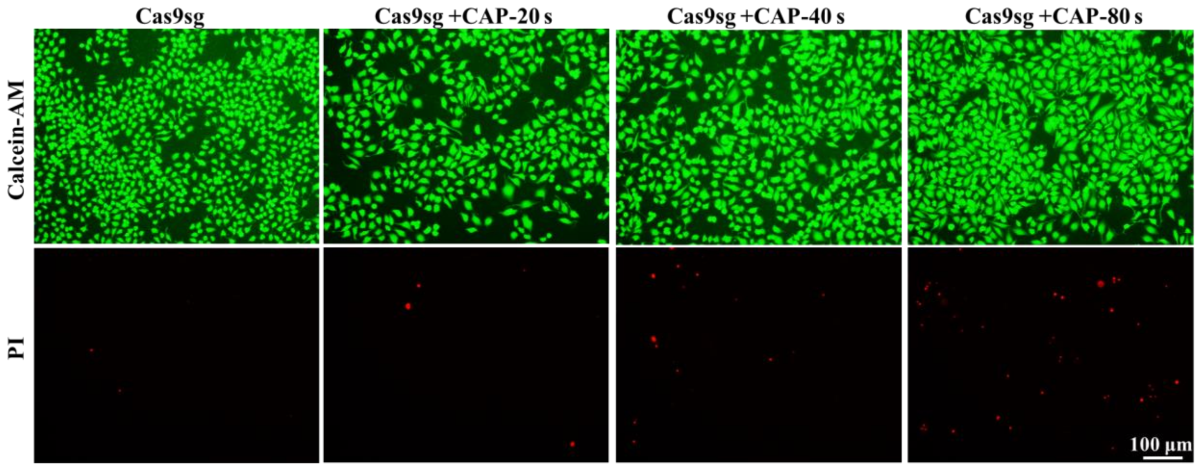

Figure S6. Fluorescence images of MCF-7 cells for 24 h after the CAP treatment. The viable cells are stained green with calcein-AM, and dead cellsare stained red with PI. The scale bar indicates 100 μm.

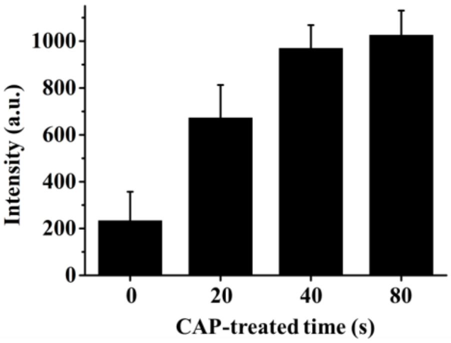

Figure S7. Fluorescence intensity of MCF-7 cells treated with Cas9sg-488 for 24 h after CAP exposure for various time periods. The data are shown as mean ± SD (n = 3).

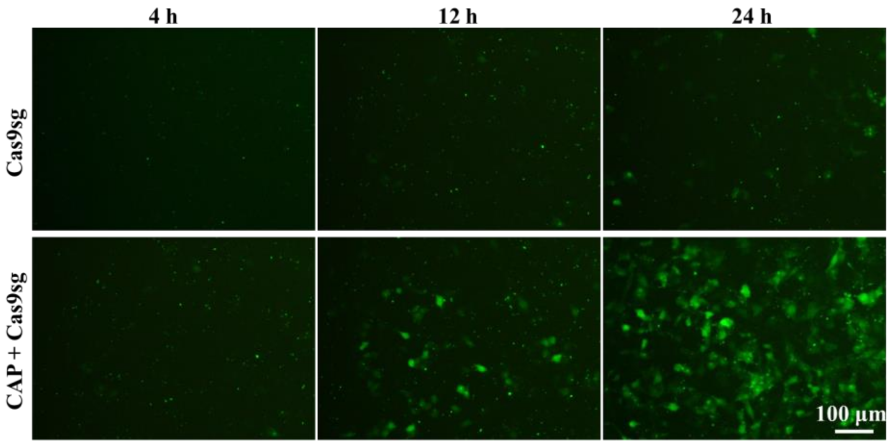

Figure S8. Fluorescence imaging of the MCF-7 cells treated with Cas9sg-488 for different time intervals after CAP exposure for 80 s. The scale bar indicates 100 μm.

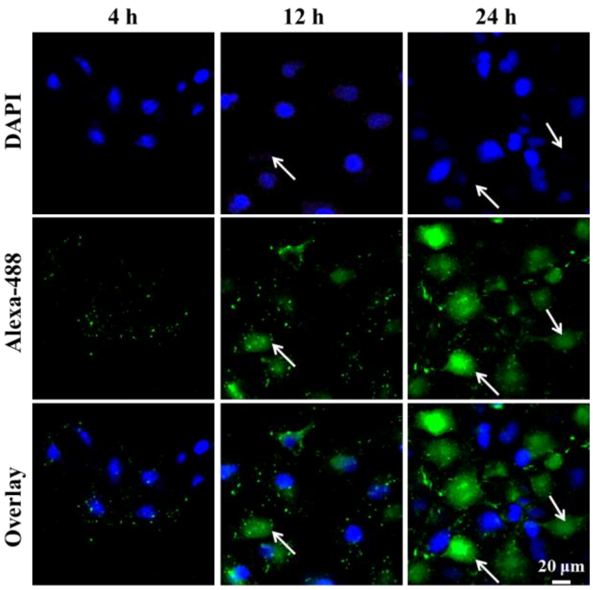

Figure S9. Confocal fluorescence imaging of MCF-7 cells treated with Cas9sg-488 for different time intervals after CAP exposure for 80 s. Blue and green fluorescence images show nuclear staining with DAPI and Alexa-488, respectively. White arrows indicate that the fluorescence signals from nuclei stained with DAPI are relatively weak in cells with efficient nuclear import of Cas9sg-488. The scale bar indicates 20 μm.

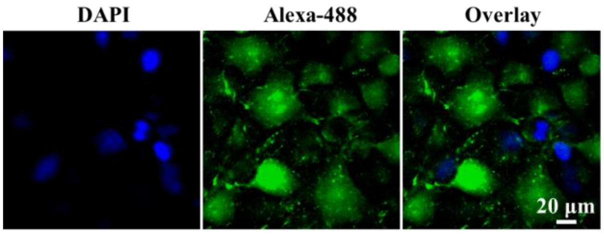

Figure S10. Confocal fluorescence imaging of MCF-7 cells treated with Cas9sg-488 for different time intervals *via* Lipofectamine transfection. The scale bar indicates 20 μm.

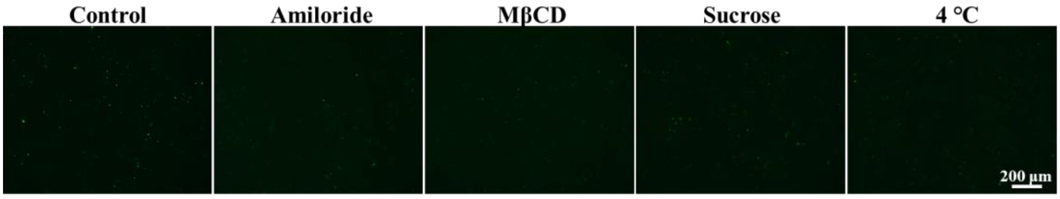

Figure S11. Fluorescence images of Cas9sg-488 uptake by the MCF-7 cells pre-treated with different endocytosis inhibitors and at a low temperature (4 ºC) ($C_{Cas9sg\text{-}488}$ = 8 nmol L^{-1}). The scale bar indicates 200 μm.

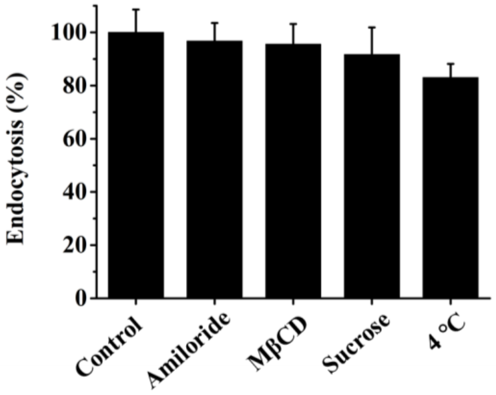

Figure S12. Effects of different endocytosis inhibitors and low temperature of 4 °C on cellular uptake of Cas9sg-488 in MCF-7 cells without CAP treatment ($C_{Cas9sg-488}$ = 8 nmol L^{-1}). The data are shown as mean ± SD (*n* = 3).

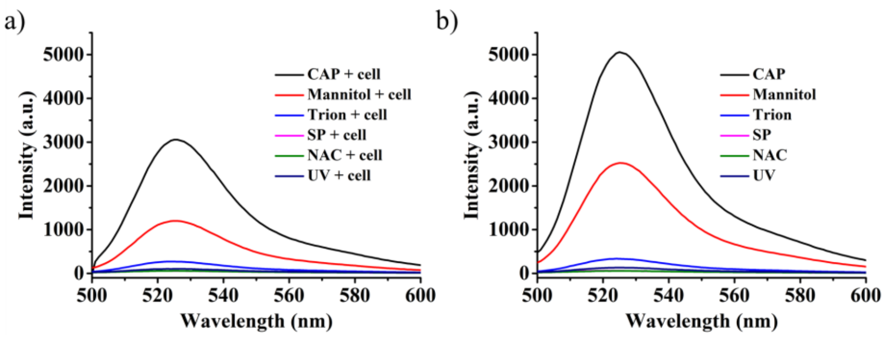

Figure S13. Fluorescence intensity of DCFDA probes in the media (a) with MCF-7 cells and (b) without MCF-7 cells after the CAP treatment under different RONS scavengers and UV radiation.

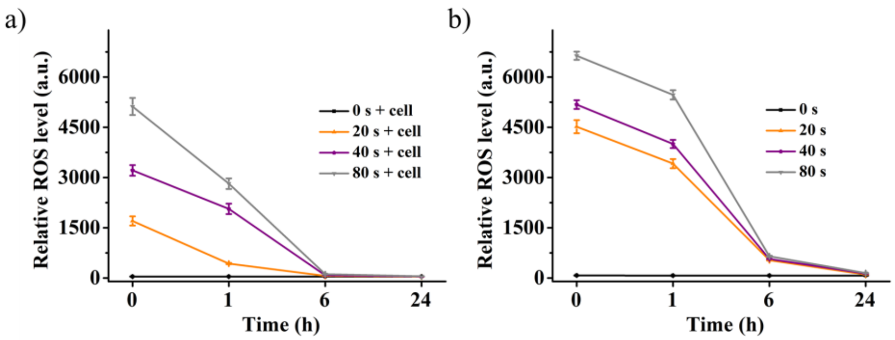

Figure S14. Relative RONS levels in the media (a) with MCF-7 cells and (b) without MCF-7 cells after the CAP treatment. The data are shown as mean ± SD (*n* = 3).

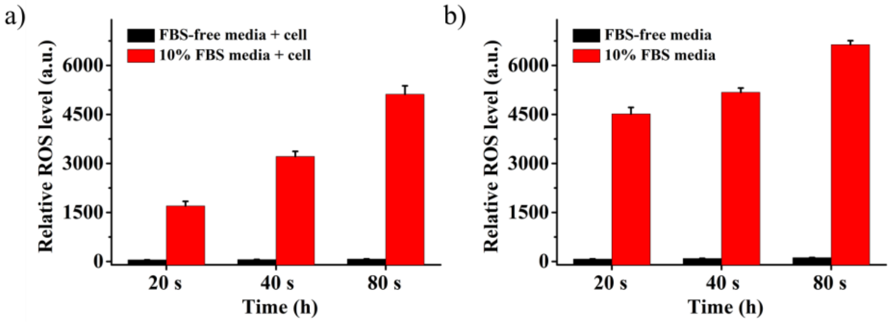

Figure S15. Relative RONS levels in the media (with or without FBS) (a) with MCF-7 cells and (b) without MCF-7 cells after the CAP treatment. The data are shown as mean ± SD (*n* = 3).

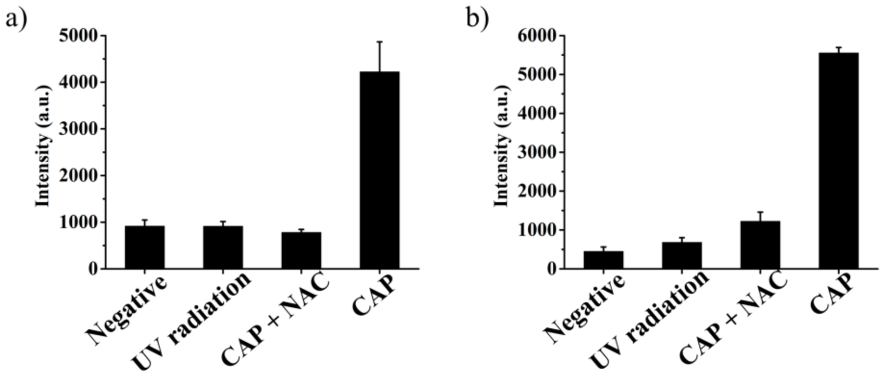

Figure S16. Intracellular (a) ROS and (b) RNS generation after the CAP treatment under UV radiation alone and in the presence of NAC. The data are shown as mean ± SD (n = 3).

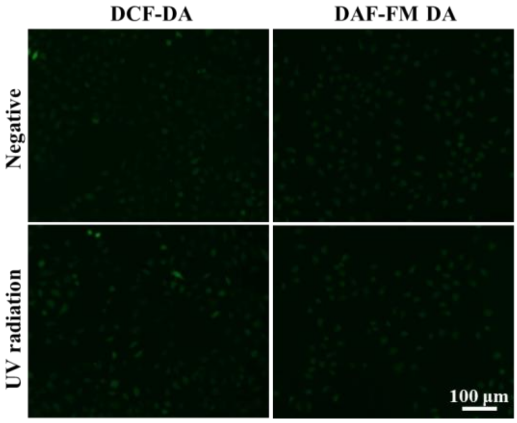

Figure S17. Intracellular ROS and RNS generation after the CAP treatment under UV radiation alone. The data are shown as mean ± SD (n = 3).

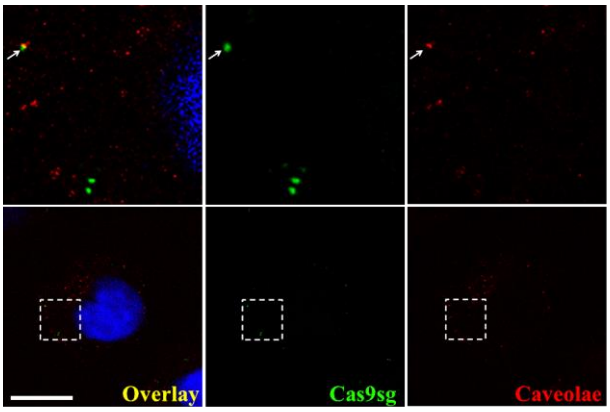

Figure S18. Confocal immunofluorescence images of co-localization of Cas9sg (green) and Caveolae (red) in the presence of NAC (C_{Cas9sg} = 8 nmol L^{-1}).

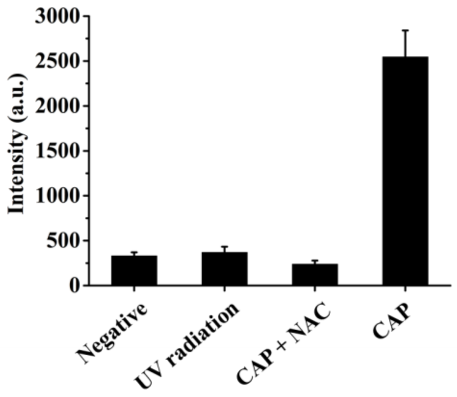

Figure S19. Intracellular Ca^{2+} levels after the CAP treatment under UV radiation alone and in the presence of NAC. The data are shown as mean ± SD (n = 3).

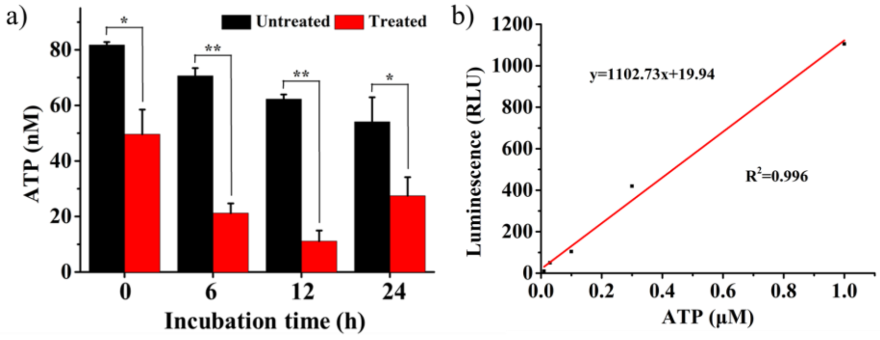

Figure S20. Determination of intracellular ATP levels after the CAP treatment: (a) Intracellular ATP levels at different time intervals after CAP treatment for 80 s, and (b) standard curves of the ATP concentration. The data are shown as mean ± SD (n = 3).

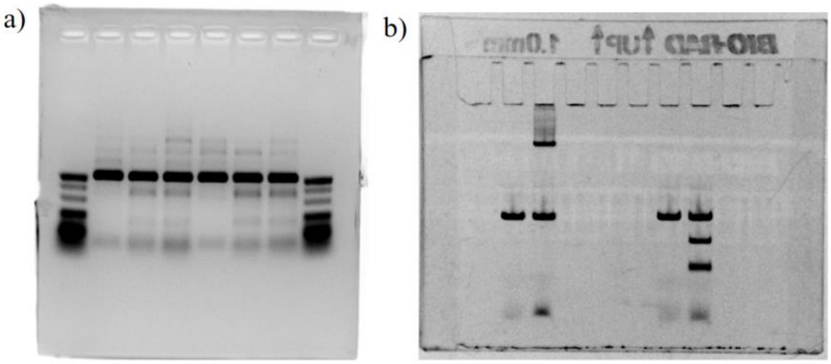

Figure S21. (a) Full WB of Figure 8c, and (b) Full WB of Figure S1.

References

[S1] Yu, X.; Liang, X.; Xie, H.; Kumar, S.; Ravinder, N.; Potter, J.; de Mollerat du Jeu, X.; Chesnut, J. D. *Biotechnol. Lett.* **2016**, *38*, 919-929.

[S2] Ha, J. S.; Byun, J.; Ahn, D. R. *Sci. Rep.* **2016**, *6*, 1–7.

[S3] Zuris, J. A.; Thompson, D. B.; Shu, Y.; Guilinger, J. P.; Bessen, J. L.; Hu, J. H.; Maeder, M. L.; Joung, J. K.; Chen, Z.; Liu, D. R. *Nat. Biotechnol.* **2015**, *33*, 73-80.

[S4] Liang, X.; Potter, J.; Kumar, S.; Zou, Y.; Quintanilla, R.; Sridharan, M.; Carte, J.; Chen, W.; Roark, N.; Ranganathan, S.; et al. *J. Biotechnol.* **2015**, *208*, 44–53.

[S5] Jacobi, A. M.; Rettig, G. R.; Turk, R.; Collingwood, M. A.; Zeiner, S. A.; Quadros, R. M.; Harms, D. W.; et al. *Methods* **2017**, *121–122*, 16-28.
